# Development of novel hybridized models for urban flood susceptibility mapping

**DOI:** 10.1038/s41598-020-69703-7

**Published:** 2020-07-31

**Authors:** Omid Rahmati, Hamid Darabi, Mahdi Panahi, Zahra Kalantari, Seyed Amir Naghibi, Carla Sofia Santos Ferreira, Aiding Kornejady, Zahra Karimidastenaei, Farnoush Mohammadi, Stefanos Stefanidis, Dieu Tien Bui, Ali Torabi Haghighi

**Affiliations:** 1 Geographic Information Science Research Group, Ton Duc Thang University, Ho Chi Minh City, Viet Nam; 2 Faculty of Environment and Labour Safety, Ton Duc Thang University, Ho Chi Minh City, Viet Nam; 3 Water, Energy and Environmental Engineering Research Unit, University of Oulu, Oulu, Finland; 4 Geoscience Platform Research Division, Korea Institute of Geoscience and Mineral Resources (KIGAM), 124, Gwahak-ro, Yuseong-gu, Daejeon, 34132 Republic of Korea; 5 Division of Science Education, Kangwon National University, Chuncheon-si, Gangwon-do 24341 Republic of Korea; 6 Department of Physical Geography and Bolin Centre for Climate Research, Stockholm University, Stockholm, Sweden; 7 Department Water Resources Engineering and Center for Middle Eastern Studies, Lund University, Lund, Sweden; 8 Research Centre for Natural Resources, Environment and Society (CERNAS), Polytechnic Institute of Coimbra, Agrarian School of Coimbra, Coimbra, Portugal; 9 Spatial Sciences Innovators, Consulting Engineering Company, Tehran, Iran; 10 Department of Arid and Mountainous Regions Reclamation, Faculty of Natural Resources, University of Tehran, Tehran, Iran; 11 Laboratory of Mountainous Water Management and Control, Faculty of Forestry and Natural Environment, Aristotle University of Thessaloniki, Thessaloniki, Greece; 12 Institute of Research and Development, Duy Tan University, 550000 Da Nang, Viet Nam; 13 Geographic Information System group, Department of Business and IT, University of South-Eastern Norway, 3800 Bø i Telemark, Norway

**Keywords:** Hydrology, Environmental impact

## Abstract

Floods in urban environments often result in loss of life and destruction of property, with many negative socio-economic effects. However, the application of most flood prediction models still remains challenging due to data scarcity. This creates a need to develop novel hybridized models based on historical urban flood events, using, e.g., metaheuristic optimization algorithms and wavelet analysis. The hybridized models examined in this study (Wavelet-SVR-Bat and Wavelet-SVR-GWO), designed as intelligent systems, consist of a support vector regression (SVR), integrated with a combination of wavelet transform and metaheuristic optimization algorithms, including the grey wolf optimizer (GWO), and the bat optimizer (Bat). The efficiency of the novel hybridized and standalone SVR models for spatial modeling of urban flood inundation was evaluated using different cutoff-dependent and cutoff-independent evaluation criteria, including area under the receiver operating characteristic curve (AUC), Accuracy (A), Matthews Correlation Coefficient (MCC), Misclassification Rate (MR), and F-score. The results demonstrated that both hybridized models had very high performance (Wavelet-SVR-GWO: AUC = 0.981, A = 0.92, MCC = 0.86, MR = 0.07; Wavelet-SVR-Bat: AUC = 0.972, A = 0.88, MCC = 0.76, MR = 0.11) compared with the standalone SVR (AUC = 0.917, A = 0.85, MCC = 0.7, MR = 0.15). Therefore, these hybridized models are a promising, cost-effective method for spatial modeling of urban flood susceptibility and for providing in-depth insights to guide flood preparedness and emergency response services.

## Introduction

Floods are the most widespread and damaging natural disaster, imposing a range of adverse effects across countries and regions all over the world^1^. These include fatalities, displacement of people, damage to infrastructure, and environmental damage, thus affecting economic activities and development^2,3^. In 2006–2015, floods were the leading cause of disaster deaths in African regions, in Central and South America and in Central, South^4^, and West Asia^5^. In 2016, worldwide floods affected ~ 78 million people and caused 4,731 fatalities and damage costing 60 billion USD^5^. Asia is the most vulnerable continent^6^, with floods being responsible for ~ 90% of all human losses due to natural hazards on that continent^7,8^.

Urban floods often cause loss of life and other health impacts such as damaging the living environment, pollution of drinking water, and outbreaks of diseases such as hepatitis E, gastrointestinal disease, and leptospirosis^9,10^. Floods in urban areas can be triggered by intensive rainfall, rapid snowmelt, and rises in water level (e.g., sea, river, lake, and groundwater)^11,12^. Also, physical location, rapid urban expansion, and land-use change have a significant effect on flood occurrences^4^. Although the extensive implementation of flood control measures, such as widespread grey infrastructures (e.g., dams and channelization) and nature-based solutions (e.g., wetlands and bioswales), most cities around the world remain vulnerable to flood hazards^13^. Low capacity of urban drainage systems can increase the inundation area and decrease the efficiency of infrastructures, and consequently, exacerbate the risk of urban floods^3^. Climate change and urbanization may increase the frequency and magnitude of urban flood events.

The frequency of flood-related disasters is projected to increase in the future due to the growing global population, particularly in developing countries, increased soil sealing as a result of urbanization, and climate change^14,15^. In Europe, for example, floods due to climate change are expected to affect three-fold more people in 2050 than in the current climate and to cause damage costing €20–40 billion^16^. Lack of financial resources, particularly in developing countries, may represent an additional challenge in flood prevention and preparation for floods^17^.

Since floods are a natural phenomenon that cannot be prevented (2007/60/EC), implementation of flood management strategies is necessary. Flood reduction, one of the key societal challenges of this century^15^, relies on prior flood risk assessment, which includes flood hazard maps showing potentially affected areas under different flood scenarios (i.e., frequency and magnitude). Several methodologies based on numerical models (statistical relationships between hydrological input and output) and physically-based models (relationships between rainfall and runoff) and techniques (e.g., remote sensing and geographic information system, GIS) have been used to generate flood hazard maps in urban areas^8,18^. However, the accuracy of these maps is affected by (i) nonlinear dynamic characteristics of floods, as a result of distinct factors such as precipitation and human activities^19^; (ii) limitations in data availability, including detailed hydrological and hydraulic data, particularly in developing countries, despite advances provided by new remote sensing techniques, such as satellites, multisensory systems, and/or radar^11^; and (iii) limited applicability of methods at different scales^20^. These drawbacks have encouraged the use of advanced data-driven models, e.g., machine learning (ML), a field of artificial intelligence that uses computer algorithms to analyze and predict information through learning from training data^21^.

In recent years, ML has been used by hydrologists for flood susceptibility mapping^11^, e.g., based on novel algorithms to improve river discharge estimation^22^, or new models built upon past flood inventory maps and including a number of flood conditioning factors^19^. Flood inundation is significantly influenced by a wide range of factors such as slope^23^, land use^10^, distance from river^3^, drainage-system capacity^11^, distance to channel^11^, and land subsidence^6,24^; therefore, ML models should be able to identify relationships between flood inundation events and these factors. Some of the most popular methods for flood susceptibility mapping are Decision Trees^25^, Artificial Neural Networks^10^, and Support Vector Machines (SVM)^19^. Among these methods, SVM is becoming increasingly well-known^19^. It is a class of support vector, a search algorithm using statistical learning theory, which is used to minimize over-fitting and reduce the expected error of learning machines^26^. It has also been extended as a regression tool, called Support Vector Regression (SVR)^27^. SVMs are suitable for both linear and nonlinear classification and are efficient and reliable tools for producing flood susceptibility maps in a GIS environment^19^.

Machine learning methods show better performance and provide more cost-effective flood susceptibility assessments than numerical and physical methods^22,26^. However, their performance may differ from one region to another, due to different geo-environmental factors^8^, the complex algorithms they contain, which sometimes make interpretation difficult^26^, and/or their structural limitations, such as the requirement for a large number of parameters, which can impair wider applications and compromise model performance^3^. Therefore, a major trend in advancing flood susceptibility predictions based on the determination of flood-prone regions is hybridization, i.e., integration of two or more ML methods, such as Adaptive Neuro-Fuzzy Interface Systems (ANFIS)^8^, integration between ML and more conventional methods, and/or soft computing techniques^26^. Novel hybrid methods involving SVR, such as ANFIS-SVR^20^, Recurrent Neural Network (RNN)-SVR^28^, Hydrologic Engineering Center-Hydrologic Modeling System (HEC-HMS)-SVR^29^, and SVR-Discrete Wavelet Transform (DWT)-Empirical Mode Decomposition (EMD)^30^, have achieved notable progress in terms of accuracy, generalization, uncertainty, performance, and robustness.

Some ML models have been applied in different watersheds (i.e., natural areas) around the world^3,8,11^, but urban flood susceptibility modeling still remains challenging. The aim of this study was to develop new hybridized models for urban flood susceptibility mapping by integrating SVR with two metaheuristic optimization algorithms: (i) Bat and Grey Wolf Optimizer (GWO) and (ii) the wavelet transform concept. As a study case, the novel hybridized models (Wavelet-SVR-Bat and Wavelet-SVR-GWO) and standalone SVR model were applied to Amol city, in northern Iran, and their results were compared using different cutoff-dependent and cutoff-independent evaluation criteria. Production of accurate flood susceptibility maps is essential to support decision-making on developing resilience planning, emergency responses, and flood mitigation.

### Description of the study area

**Figure 1:**
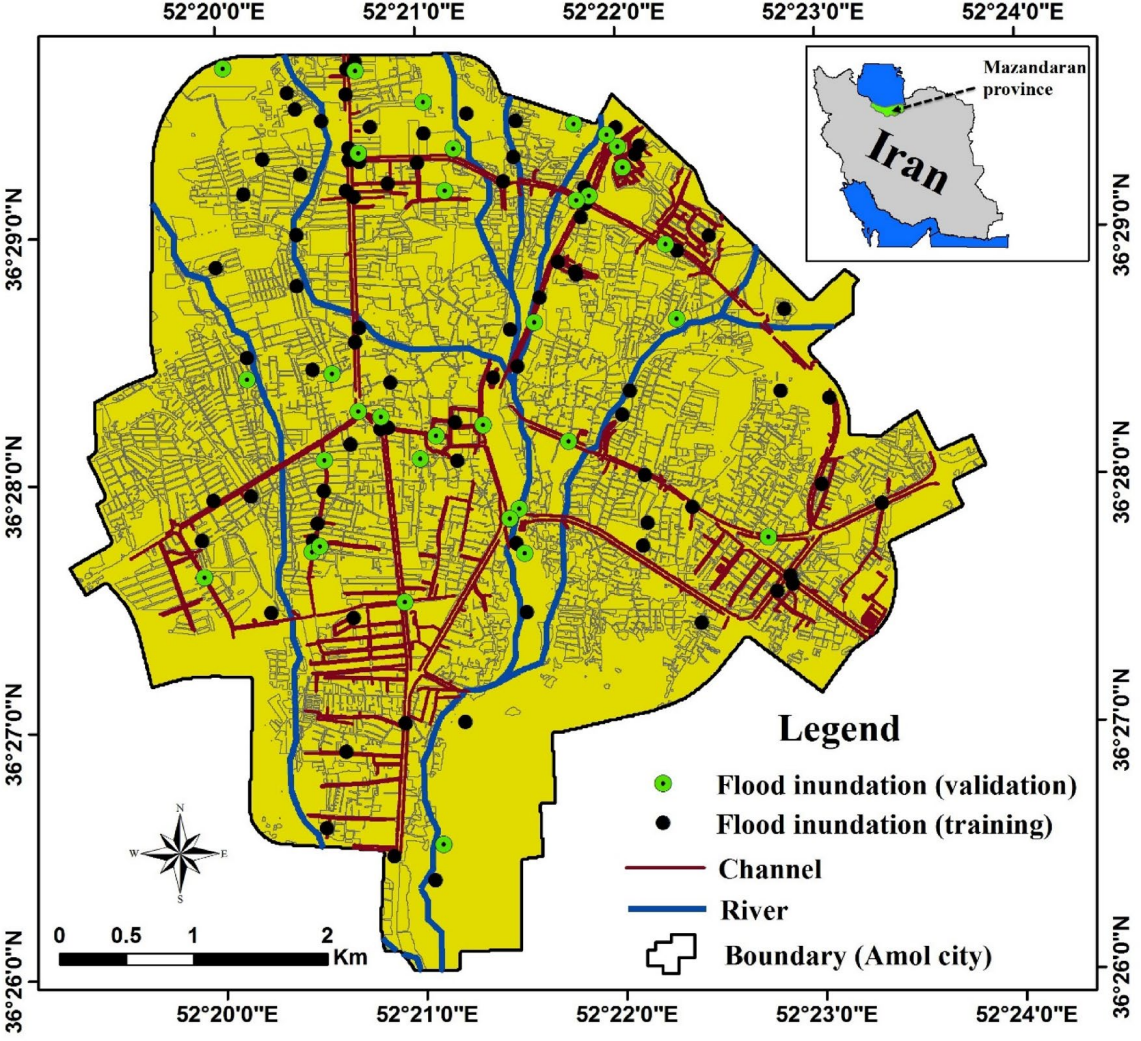
Location of Amol city, Mazandaran province, Iran, and sites affected by past floods. The map was generated using ArcGIS Desktop 10.7.1, https://desktop.arcgis.com/en.

**Figure 2:**
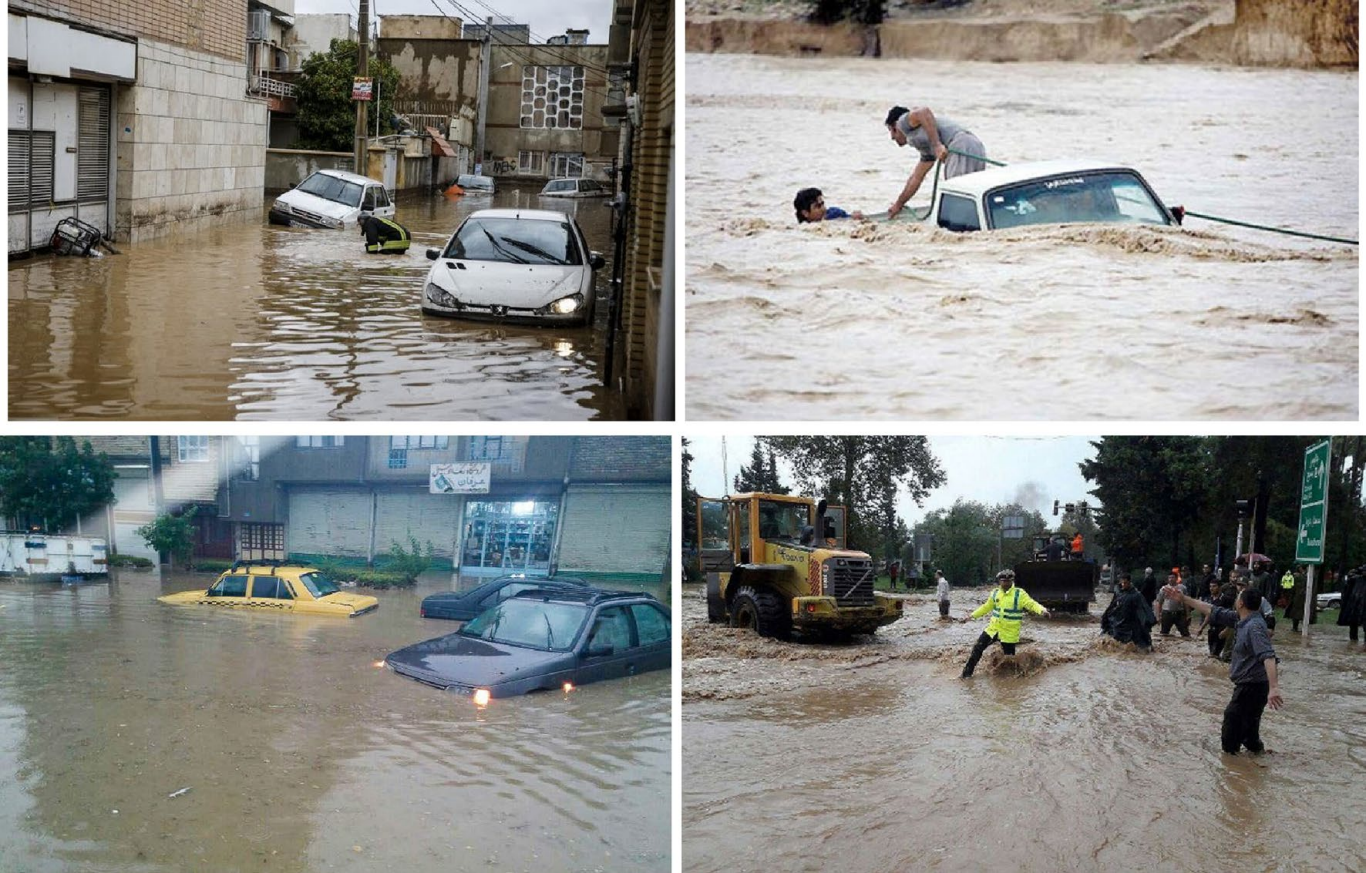
Field photographs of flooded sites in Amol city, taken on March 17, 2019 (photographs by Hamid Darabi).

The city of Amol (36°26′–36°29.5′N, 52°19.6′–52°24′E) is located on the southern coast of the Caspian Sea, in the west of Mazandaran Province, northern Iran (Fig. 1). The city lies near the outlet of the Haraz River watershed (about 1,100 km^2^ in area), with the Haraz river passing through the heart of Amol city and then reaching the Caspian Sea^31^. Amol is a rapidly growing and heavily industrialized city, currently occupying an area of 27.1 km^2^ and hosting a population of approximately 300,000. Residential areas in the city are mainly surrounded by alluvial plains (agricultural land, orchards) to the north and high mountains (the Alborz range) covered by forest to the south^32^. The region has a humid climate, with a moderate temperature (17.4 °C mean annual temperature) and mean annual precipitation of 650 mm (2001–2019), recorded at Amol weather station of the Iranian Meteorological Organization (IRIMO). Amol city was selected for the present study due to the many catastrophic floods that have occurred annually in recent years, damaging thousands of homes and infrastructure, disrupting traffic, trade, and public services, and taking lives. Figure 2 shows some consequences of previous floods in Amol city.

## Results

### Multicollinearity analysis

After implementing the variance inflation factors (VIF) and tolerance (TOL) indices, it was found that there is no critical multicollinearity between the factors, since the values were all placed within the acceptable range (Table 1).

**Table 1:** Variance inflation factor (VIF) and tolerance (TOL) values representing the multicollinearity between selected flood-controlling factors.

Flood-controlling factor	Collinearity Statistics		Std. error
	TOL	VIF	
Elevation	0.624	1.603	0
Curve number	0.987	1.013	0.002
Distance from river	0.337	2.969	0
Distance from channel	0.445	2.245	0
Precipitation	0.673	1.486	0
Slope percentage	0.919	1.089	0

### Goodness-of-fit and predictive performance of the models

#### Cutoff-independent criteria (AUC method)

**Figure 3:**
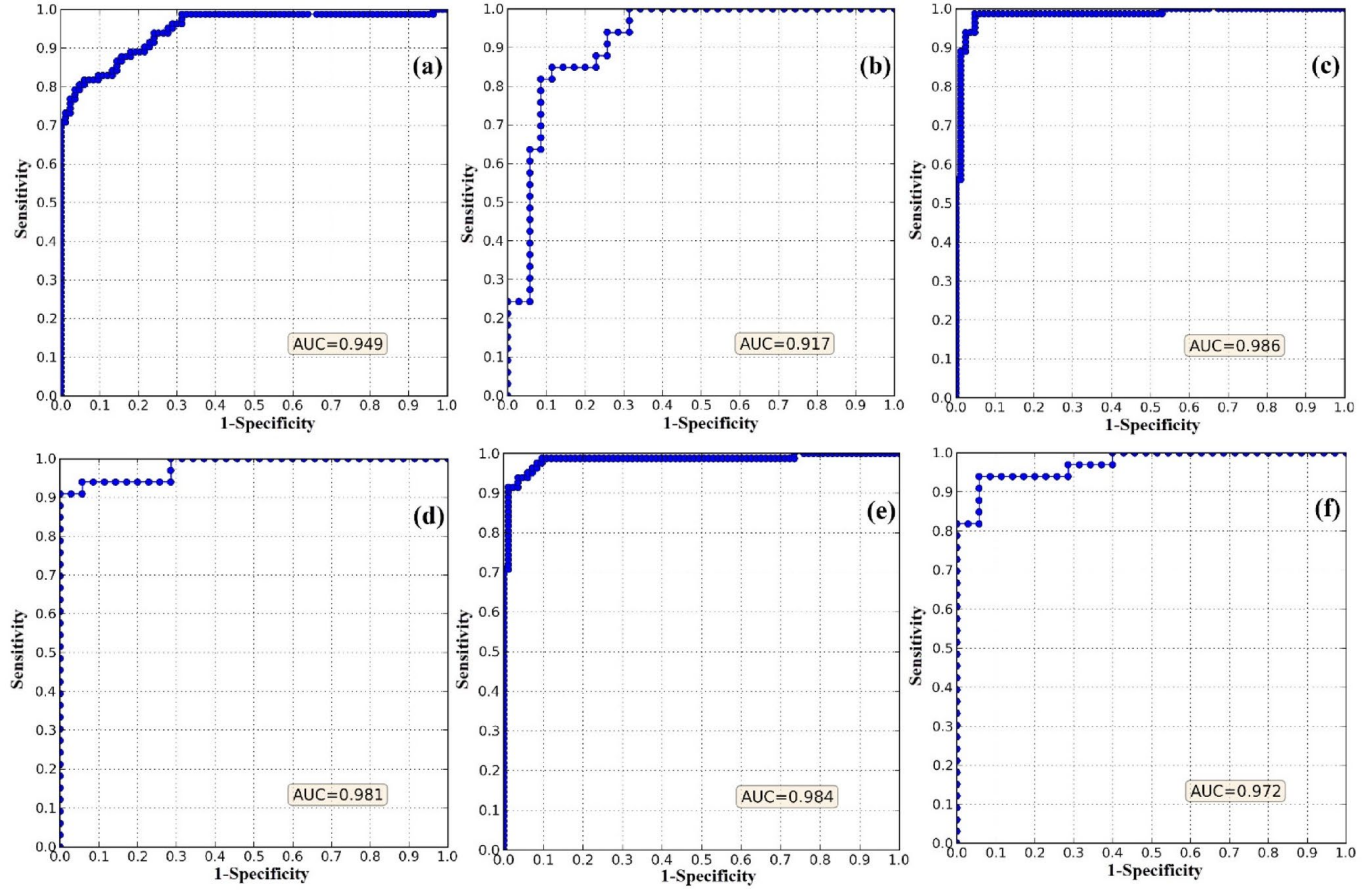
Goodness-of-fit and predictive performance of models based on the AUC metric: (**a**) SVR in training, (**b**) SVR in validation, (**c**) Wavelet-SVR-Bat in training, (**d**) Wavelet-SVR-Bat in validation, (**e**) Wavelet-SVR-GWO in training, and (**f**) Wavelet-SVR-GWO in validation.

The results of goodness-of-fit (i.e., accuracy in the training step) and predictive performance (i.e., accuracy in the validation step) of the three models in terms of the AUC metric are displayed in Fig. 3. Based on the AUC, Wavelet-SVR-Bat had the highest accuracy in training (AUC = 0.986), followed by Wavelet-SVR-GWO (AUC = 0.984) and the standalone SVR (AUC = 0.949). The goodness-of-fit of each model was measured based on the data used to calibrate the model, and it shows how well the model fits the training dataset. The predictive power of the model cannot be judged using the goodness-of-fit of the model, so a separate analysis of predictive performance was conducted. In terms of predictive performance, Wavelet-SVR-GWO had the highest accuracy (AUC = 0.981), slightly better than Wavelet-SVR-Bat (AUC = 0.972). The AUC value of the standalone SVR model in this step was 0.917. Therefore, in order of performance, the ranking was: Wavelet-SVR-GWO, Wavelet-SVR-Bat, and SVR, although with minor differences.

#### Cutoff-dependent criteria

The results of accuracy assessment using the cutoff-dependent criteria TPR, FPR, Accuracy, MCC, F-score, and MR, are summarized in Table 2. These evaluation metrics provided better insights regarding the accuracy of the three models.

**Table 2:** Goodness-of-fit and predictive performance of models based on cutoff-dependent criteria.

Evaluation criteria	Goodness-of-fit (training step)			Predictive performance (validation step)		
	SVR	Wavelet-SVR-Bat	Wavelet-SVR-GWO	SVR	Wavelet-SVR-Bat	Wavelet-SVR-GWO
TPR (sensitivity)	0.864	0.973	0.940	0.848	0.857	1.000
FPR (1 − specificity)	0.142	0.109	0.037	0.145	0.090	0.125
Accuracy (A)	0.860	0.927	0.951	0.852	0.882	0.926
MCC	0.721	0.858	0.903	0.705	0.766	0.861
F-score	0.858	0.923	0.951	0.848	0.882	0.918
MR	0.139	0.072	0.048	0.147	0.117	0.073

*TPR* true positive rate, *FPR* false positive rate, *MCC* Matthews correlation coefficient, *MR* misclassification rate.

In terms of goodness-of-fit, Wavelet-SVR-GWO was the best model. It had a higher Accuracy (0.951), MCC (0.903), and F-score (0.951), and also a lower MR (0.048) and FPR (0.037) than other models (Table 1). However, in terms of the TPR metric, Wavelet-SVR-Bat (0.973) was slightly better than Wavelet-SVR-GWO (0.94). Importantly, the standalone SVR had the lowest accuracy based on all cutoff-dependent evaluation metrics. As indicated above, goodness-of-fit only provides insights regarding the degree of fit of data to the model. Therefore, the prediction capability of the models was investigated in a validation step. The results of the validation analysis clearly indicated the superior performance of Wavelet-SVR-GWO based on the TPR (1.00), Accuracy (0.926), MCC (0.861), F-score (0.918), and MR (0.073). It was followed by Wavelet-SVR-Bat, which performed very well in terms of TPR (0.857), Accuracy (0.882), MCC (0.766), F-score (0.882), and MR (0.117). The standalone SVR had the lowest accuracy in terms of predictive performance, with TPR of 0.848, FPR of 0.145, Accuracy of 0.852, MCC of 0.705, F-score of 0.848, and MR of 0.147. In order of performance, the ranking was again: Wavelet-SVR-GWO, Wavelet-SVR-Bat, and SVR.

#### RMSE metric

**Figure 4:**
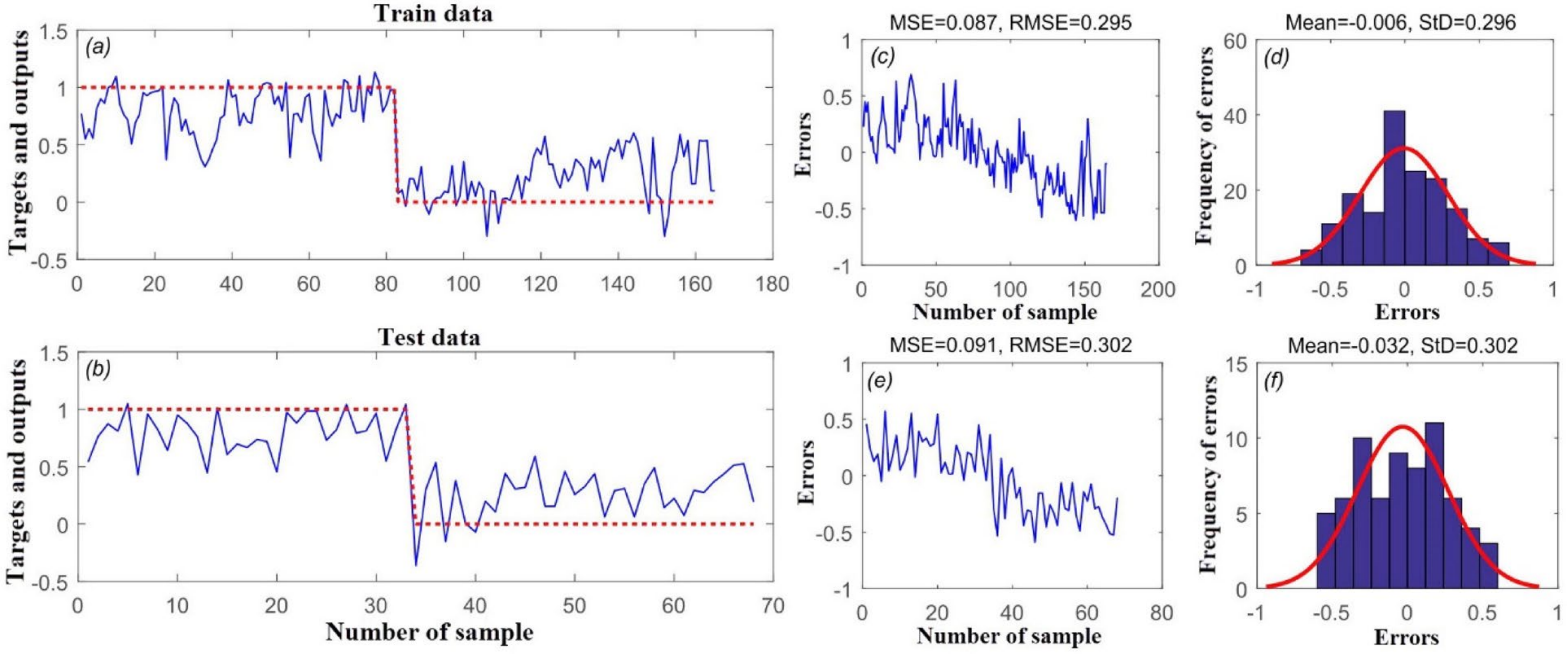
(**a**) Target and output SVR value of training data samples, (**b**) target and output SVR value of testing data samples, (**c**) mean squared error (MSE) and RMSE value of training data samples, (**d**) frequency of errors for training data samples, (**e**) MSE and RMSE value of testing data samples, and (**f**) frequency of errors for testing data samples.

Figure 4 shows the target and outputs of the SVR model for the (a) training and (b) validation datasets, and (c, e) the mean squared error (MSE) and root mean square error (RMSE) values for these datasets. As can be seen, MSE and RMSE values of 0.087 and 0.295 were obtained for SVR in predicting the training set, while MSE and RMSE values of 0.091 and 0.302 were obtained in predicting the validation set. Mean frequency of errors for the training and validation datasets was 0.006 and 0.032, respectively (Fig. 4d,f).

**Figure 5:**
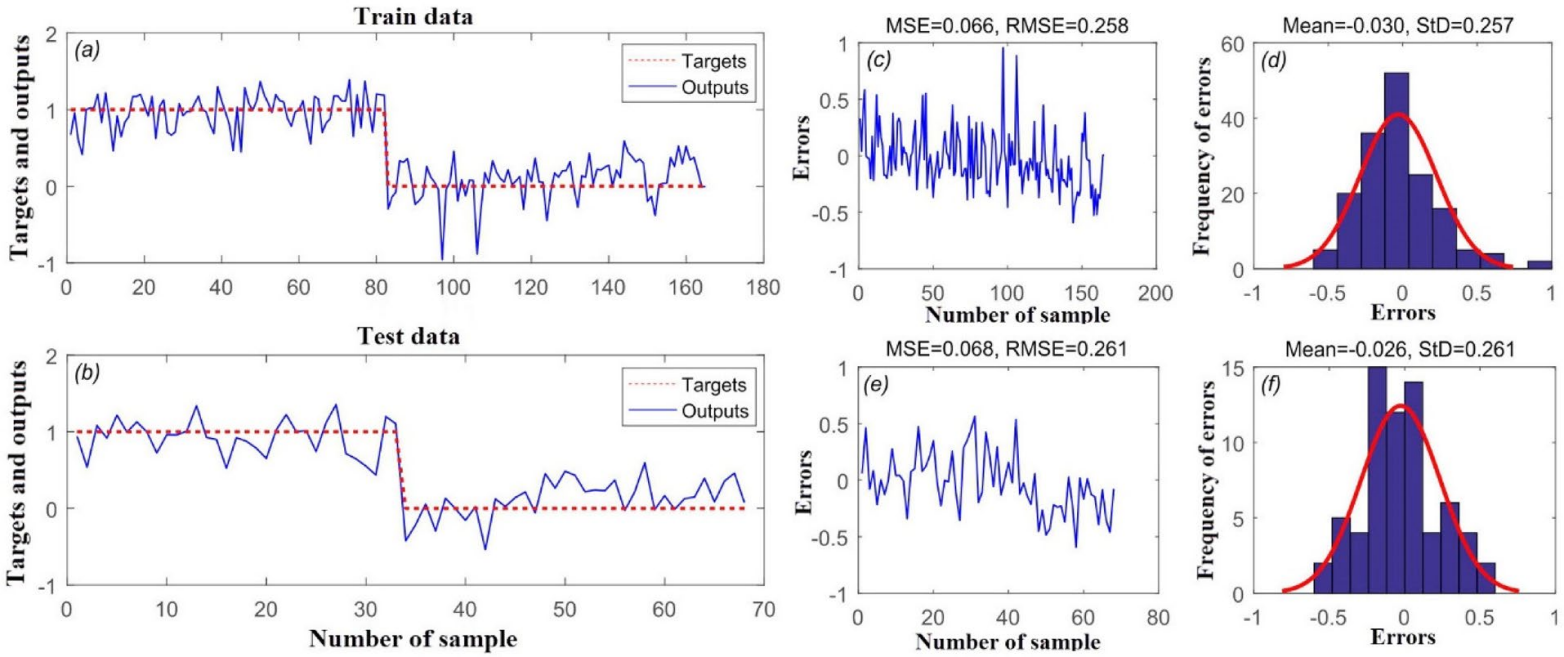
(**a**) Target and output Wavelet-SVR-BAT value of training data samples, (**b**) target and output Wavelet-SVR-BAT value of testing data samples, (**c**) mean squared error (MSE) and root mean square error (RMSE) of training data samples, (**d**) frequency of errors for training data samples, (**e**) MSE and RMSE value of testing data samples, and (**f**) frequency of errors for testing data samples.

Figure 5 shows the accuracy of the Wavelet-SVR-BAT model based on the MSE and RMSE metrics. As can be seen from Fig. 5c, MSE and RMSE values of 0.066 and 0.258 were obtained for Wavelet-SVR-BAT in predicting the training set, while it had MSE and RMSE values of 0.068 and 0.251 in the validation step (Fig. 5e). Mean frequency of errors for the training and validation datasets was 0.030 and 0.026, respectively (Fig. 5d,f).

**Figure 6:**
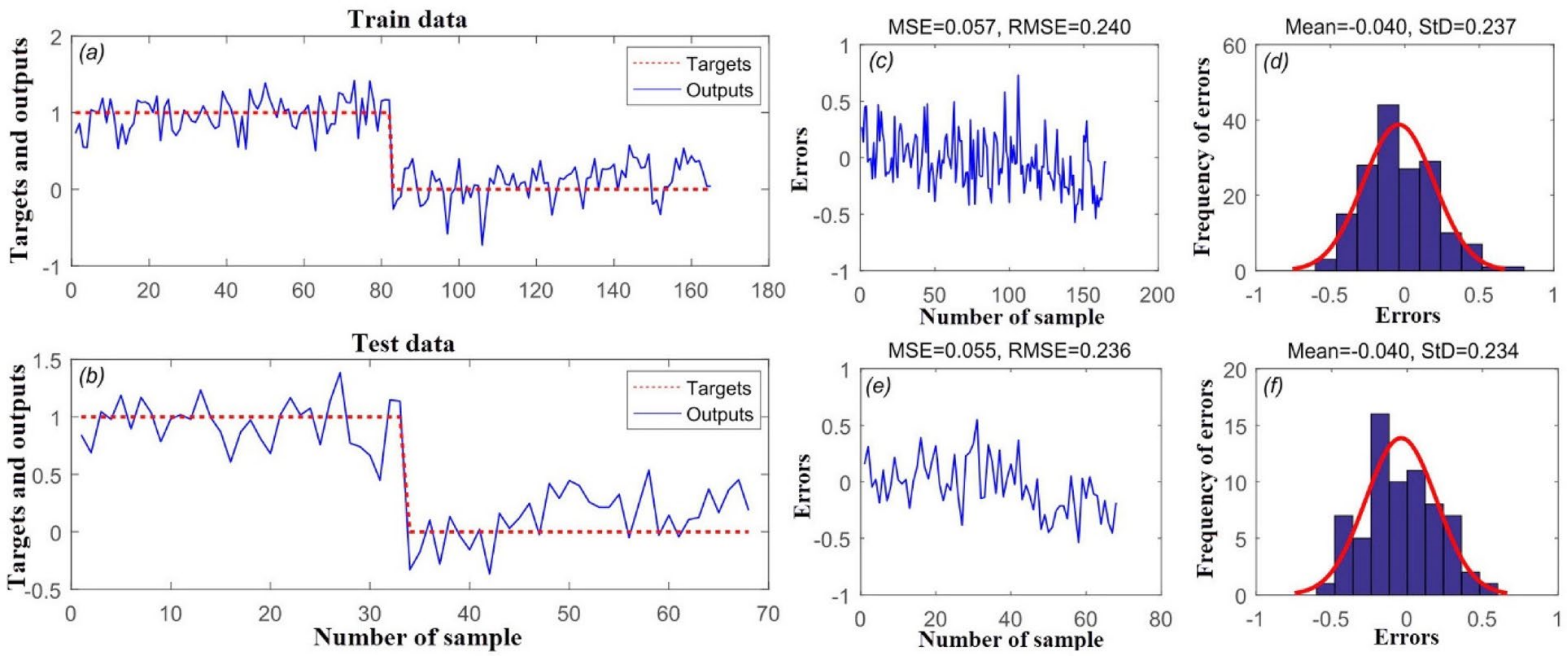
(**a**) Target and output Wavelet-SVR-GWO value of training data samples, (**b**) target and output Wavelet-SVR-GWO value of testing data samples, (**c**) mean squared error (MSE) and root mean square error (RMSE) value of training data samples, (**d**) frequency of errors for training data samples, (**e**) MSE and RMSE value of testing data samples, and (**f**) frequency of errors for testing data samples.

Figure 6 indicates the goodness-of-fit and predictive performance of the Wavelet-SVR-GWO model based on the MSE and RMSE metrics. As shown in Fig. 6c,e, Wavelet-SVR-GWO had MSE and RMSE values of 0.057 and 0.240 in the training step, and MSE and RMSE values of 0.055 and 0.236 in predicting the validation set. Mean frequency of errors for the training and validation datasets was 0.040, and 0.040, respectively (Fig. 6d,f).

According to the results, Wavelet-SVR-GWO and Wavelet-SVR-BAT had lower MSE and RMSE values than the standalone SVR model. Therefore, Wavelet-SVR-GWO can be regarded as the best model (of the three) for spatially modeling flood susceptibility in urban areas.

### Urban flood susceptibility maps

**Figure 7:**
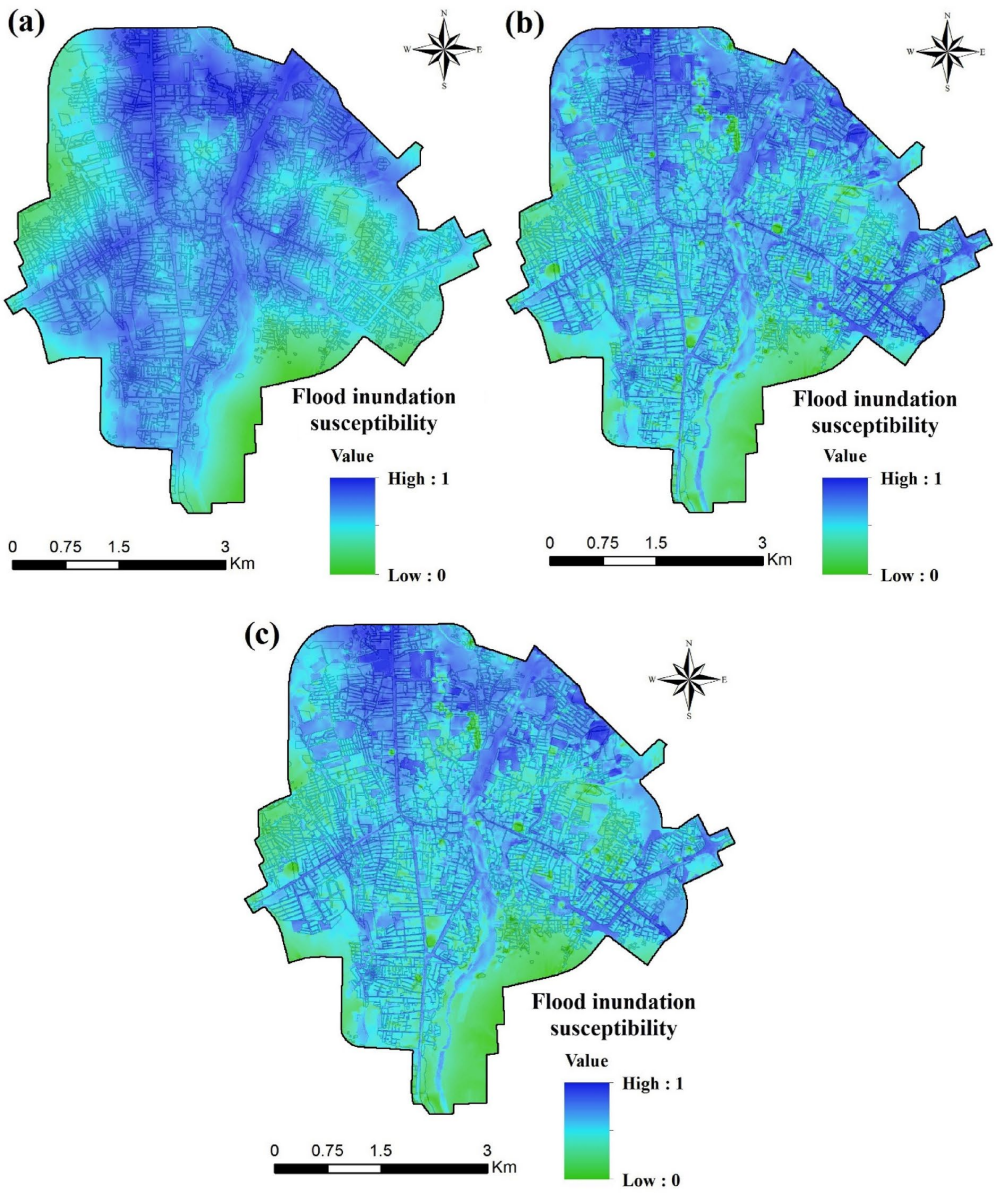
Flood inundation susceptibility maps for the city of Amol according to: (**a**) the SVR model, (**b**) the Wavelet-SVR-Bat model, and (**c**) the Wavelet-SVR-GWO model. These maps were generated using ArcGIS Desktop 10.7.1, https://desktop.arcgis.com/en/.

Figure 7 shows the urban flood susceptibility maps for Amol city obtained using the SVR, Wavelet-SVR-Bat, and Wavelet-SVR-GWO models. In this example, urban flood susceptibility is used as a reference to estimate the risk of an area being inundated in any one year in the future. Areas depicted in blue have higher inundation susceptibility, whereas areas in green have lower, with higher susceptibility indicating a higher spatial potential for inundation. The maps of the study area generated by the hybridized models (Fig. 7b,c) showed a similar overall spatial pattern, with high flood probability in the northern part of the study area. However, there were apparent differences between the results of hybridized models and the standalone SVR, which showed higher flood susceptibility affecting larger areas, including those surrounding the river and the channels dispersed within the city. According to the SVR model, 11.7% of the city falls in the very high flood inundation susceptibility class, whereas with Wavelet-SVR-Bat and Wavelet-SVR-GWO models this class covers only 3.4% and 2.4%, respectively, of Amol (Table 3). However, all the models showed high flood susceptibility for the majority of the study area (affected area ranged from 40.7 to 62.3% with different models).

**Table 3:** Relative distribution of flood inundation classes from different models.

Model type	Model name	Flood inundation classes				
		Very low	Low	Medium	High	Very high
Standalone	SVR	2.7	11.3	29.5	44.8	11.7
Hybridized models	Wavelet-SVR-Bat	0.2	2.2	31.9	62.3	3.4
	Wavelet-SVR-GWO	0.3	10.9	45.7	40.7	2.4

**Figure 8:**
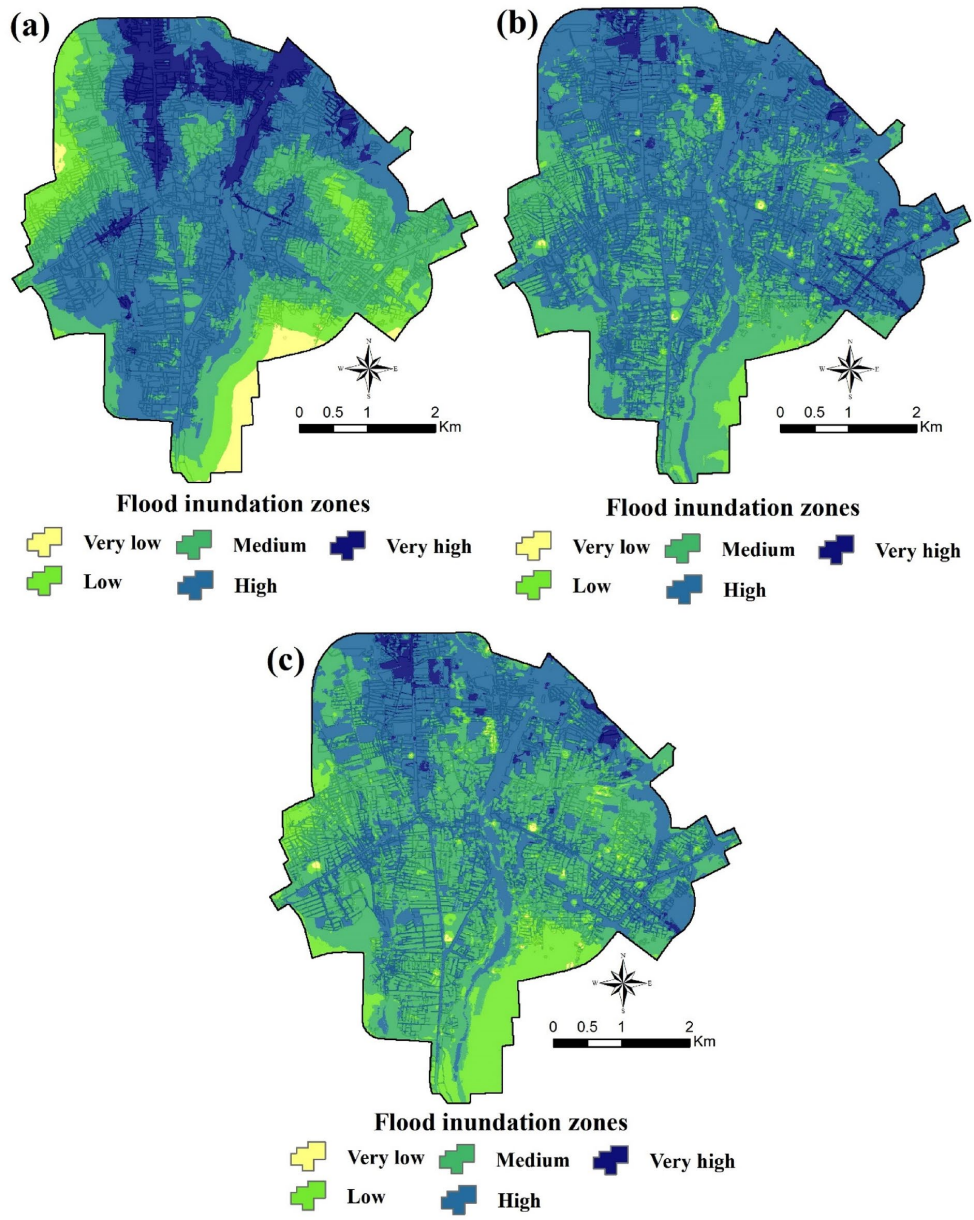
Flood inundation zone maps for the city of Amol produced by: (**a**) the SVR model, (**b**) the Wavelet-SVR-Bat model, and (**c**) the Wavelet-SVR-GWO model. These maps were generated using ArcGIS Desktop 10.7.1, https://desktop.arcgis.com/en/.

Figure 8 shows flood inundation zone maps for the city of Amol, created using the SVR, Wavelet-SVR-Bat, and Wavelet-SVR-GWO models. In the SVR model, 44.8% of the area belongs to the high flood inundation zone, 29.5% medium, 11.7% very high, 11.3% low, and 2.7% very low zones. The results of the Wavelet-SVR-Bat model indicated that the majority of the area (62.3%) belongs to the high flood inundation zone, followed by medium (31.9%), very high (3.4%), low (2.2%), and very low (0.2). The results of the Wavelet-SVR-GWO model showed that approximately 2.4%, 40.7%, 45.7%, 10.9%, and 0.3% of the study area belongs to very high, high, medium, low and very low flood inundation zones, respectively (Fig. 8c).

When the flood susceptibility maps prepared with Wavelet-SVR-GWO and Wavelet-SVR-Bat were compared with flood inundation locations in the past, it was found that there was excellent agreement between them. Two schools in Amol are located within zones with a high and very high risk of flood inundation (in the northern part of the study area), which results in children being exposed to urban flood inundation. As discussed by Alderman et al*.*^9^, child health in flooded areas must be better reflected in flood mitigation and preparedness programs.

### Comparison of model predictions

**Figure 9:**
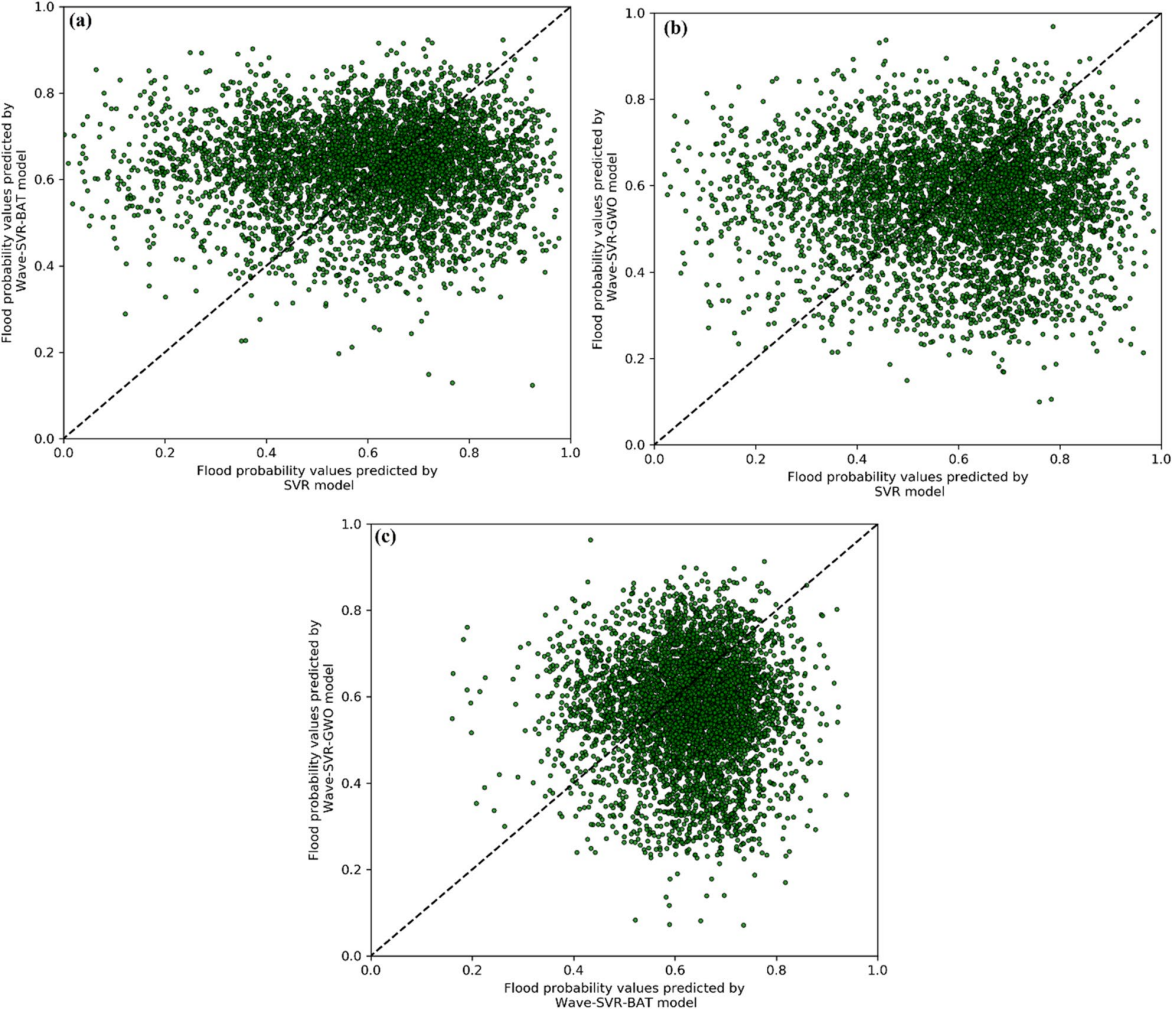
Pairwise agreement plots (PAP) showing results of pair-wise comparisons between: (**a**) SVR and Wavelet-SVR-Bat, (**b**) SVR and Wavelet-SVR-GWO, and (**c**) Wavelet-SVR-Bat and Wavelet-SVR-GWO.

In order to assess spatial agreement between the models, a pairwise agreement plot (PAP) was used. A self-explanatory graphical presentation of the results for the three models is provided in Fig. 9. Unlike a number of quantitative indices that are merely based on metaheuristic classifications and have been repeatedly used for assessing spatial agreement (e.g., kappa), PAP provides more information about the overall agreement and about the data distribution associated with such agreement or disagreement. The dataset includes all the susceptibility values derived from each spatial model, featured as points and ranging from 0 to 1. The diagonal reference line (i.e., 1:1 line) represents the degree to which the models show agreement, meaning that the closer the points get to the 1:1 line, the stronger their spatial agreement. A diagonal distribution of points close to the reference line is ideal, as it represents two perfectly identical susceptibility maps. However, it is normally not achieved since different mathematics produces different susceptibility patterns.

Since the same core mathematical architecture (i.e., SVR) was used in the three models investigated in this study, a certain degree of agreement was achieved, as expected. Figure 9a, comparing the predictions from SVR and Wave-SVR-BAT models, reveals a relatively high dispersion level between selected points. Figure 9b, comparing the SVR and Wave-SVER-GWO models, shows the highest dispersion among the models. Intuitively, these plots make some inferences regarding the divergence of hybridized algorithms from their core architecture (SVR), based on which divergence appears to be higher for the Wavelet-SVR-GWO hybrid, while Wavelet-SVR-Bat has slightly better agreement with its core. In contrast, in Fig. 9c, focusing on the susceptibility maps of the Wavelet-SVR-Bat and Wavelet-SVR-GWO models, the point cloud seems to be intensely distributed around the reference line and mainly corresponds to values ranging from 0.6 to 0.8 (less dispersed values spreading above 0.5), indicating that these two hybridized models have a stronger spatial agreement. Note that these plots do not have connotations of validity and that the inferences refer only to the spatial agreement level. The values above and below the reference line do not signify any superiority, as they may have been overestimated by one model or underestimated by the other.

### Importance of flood conditioning factors

The relative importance of flood conditioning factors based on the SVR, Wavelet-SVR-Bat, and Wavelet-SVR-GWO models is summarized in Table 4. According to all three models, distance from the channel, distance from river, and curve number are the most important flood conditioning factors in the study area. It is reasonable because most floods in the area occur near channels and rivers, where the curve number is considerable (CN > 70). Slope and elevation factors made a moderate contribution to the modeling process, based on all three models. Precipitation showed the lowest importance among all factors. This is probably due to the small spatial variation in the precipitation layer in Amol city. It is in agreement with spatial analysis of flooding events (i.e., observations) in the study area, as demonstrated in Fig. 2, which shows some examples where several factors have led to flooding. However, there were significant differences in the contribution of flood conditioning factors to the modeling process (Table 4).

**Table 4:** Overall importance of predictors in the three models used.

Excluded factor	Relative decrease in AUC (%)		
	SVR	Wavelet-SVR-Bat	Wavelet-SVR-GWO
Distance from channel	38.4 ± 3.6	39.5 ± 4.2	39.1 ± 3.9
Distance from river	31 ± 2.5	32.3 ± 3.1	33.1 ± 3.2
Curve number	26.2 ± 3.4	25.9 ± 2.8	25.7 ± 2.4
Slope	8.7 ± 3.5	8.2 ± 2.6	8.4 ± 2.8
Elevation	4.1 ± 1.9	4.6 ± 2.3	4.7 ± 2.9
Precipitation	3.6 ± 0.5	3.5 ± 0.4	3.2 ± 0.6

## Discussion

Based on the different statistical evaluation metrics employed in this study, both hybrid models tested (Wavelet-SVR-GWO and Wavelet-SVR-Bat) showed superior performance to the standalone SVR model. The results also demonstrated that Wavelet-SVR-GWO produced more trustworthy flood susceptibility maps than the other models. The GWO algorithm is flexible, robust, easy to enforce, and improves the performance of the model. Mirjalili et al*.*^33^ investigated the optimization performance of GWO and found that it has considerable capacity for optimizing models. Saxena and Shekhawat^34^ confirmed the satisfactory efficiency of GWO in optimizing SVM for an air quality classification issue. Yang^35^ describes the characteristics of the Bat algorithm that lead to its optimizing and improving model performance. Bat is a robust algorithm inspired by the echolocation behavior of bats. Yang^29^ showed that it is able to outperform other metaheuristic algorithms, such as PSO and genetic algorithm (GA), in terms of both convergence speed and improved local optima avoidance. In addition, wavelet transformers are known to be strong decomposition tools that enable prediction models to access more information at different dissected scales and dimensions of data domain^36^. Standalone models may not cope with the non-stationary properties of data, either spatially or temporally, especially when dealing with highly complicated spatial interrelationships and the problem of data scarcity (such as urban environments). Regarding the benefits of Bat and GWO algorithms and wavelet transformers, the results of this study are in agreement with previous findings^34,37^.

Information on the relative importance of flood conditioning factors (i.e., predictor variables) is of practical relevance to natural disaster managers dealing with allocating and planning limited resources for flood hazard management^8^. Kalantari et al*.*^2^ indicated that analysis of the relationship between flood events and geoenvironmental variables allows managers to focus on the influences of human activities. Although expert opinion-based methods (e.g., analytical hierarchy process (AHP)) have been applied to analyze the relative importance of geoenvironmental factors and determine their relationships with urban flood events, they are prone to subjective judgments. In fact, expert opinion-based methods determine the weight of factors based on the pairwise comparison, which can be considered a drawback. Fernández and Lutz^38^ used the AHP method for urban flood hazard zoning in Tucumán Province, Argentina, and concluded that, as flood-prone areas can be identified based on expert opinions, this method can be a useful tool if flood event data and maps are not available. Machine learning models use real flood occurrences to analyze the role of flood conditioning factors in modeling and determine the contribution of variables^39^. This approach helps in reducing bias and subjectivity in decision-making^11^. Importantly, as discussed by Khosravi et al*.*^27^, the relative importance of predictor variables to a model is probably affected by the modeling strategy (i.e., model structure, etc.). Therefore, the relative importance of factors should be investigated using at least two models. In our study, the relative importance of flood conditioning factors was analyzed based on all three models (standalone SVR, Wavelet-SVR-Bat, and Wavelet-SVR-GWO), which showed excellent performance. All models demonstrated that distance from channel made the highest contribution to urban flood modeling, followed by distance from river, and curve number. This confirms results reported by Darabi et al*.*^4^ and Falah et al.^23^, who investigated the importance of flood conditioning factors in urban areas. The urban channel network generally has low conveyance capacity, resulting in flooding and inundation events in urban areas. Rivers in the study area also have low conveyance capacity, and floods often exceed the flow capacity, so inundation occurs frequently. However, there are limitations to increasing the capacity of existing drainage channels, because of land availability in urban areas and urbanization^23^. In other words, urbanization in river/channel zones is the main cause of reduced drainage capacity, changes in hydrological and hydraulic processes, and flooding in urban areas^10^. In addition, curve number, one of the main flood conditioning factors in this study, is influenced by human activities (e.g., land-use change in urban areas or in upstream watersheds) carried out to meet various needs such as residential, agricultural, industrial, mining, and other infrastructural facilities. These pose major challenges to the sustainable growth of an area.

## Concluding remarks

Flooding poses great threats to communities and property, especially in densely-populated urban environments, where intensifying urbanization leads to severe floods by increasing the area of impermeable surfaces. Since hydrometric stations are not available in urban areas, data scarcity is the main problem in spatial modeling of flood susceptibility. This study presents two new hybridized models, wavelet-SVR-GWO and wavelet-SVR-Bat, based on historical urban flood inundation events and using metaheuristic algorithms and wavelet transformation analysis. In a case study, Wavelet-SVR-GWO showed better predictive performance (AUC = 0.981, A = 0.926, RMSE = 0.236) in flood susceptibility mapping for Amol city, Iran, than Wavelet-SVR-Bat (AUC = 0.972, A = 0.882, RMSE = 0.261). Both hybridized models showed better performance than a standalone SVR. Thus metaheuristic optimization algorithms (i.e., Bat and GWO) and wavelet transform analysis can considerably enhance the learning and predictive performance of the standalone SVR model. The coupled wavelet-optimization algorithms could be a perfect fit for data mining models to find the best solution in a high-dimensional complex problem space. In Amol city, high and very high flood susceptibility classes covered about 43% of the study area and two schools fell into these areas. Using the robust flood susceptibility maps provided by the hybridized models could improve the capability of urban systems in high and very high susceptibility classes to evacuate floodwaters and reduce negative consequences for the inhabitants, both in terms of threat to human health and damage to property. However, the hybridized models should be further tested in other urban areas to validate their performance. Additional optimization algorithms, such as GA and PSO, should also be compared with those used in the present study.

## Methodology

**Figure 10:**
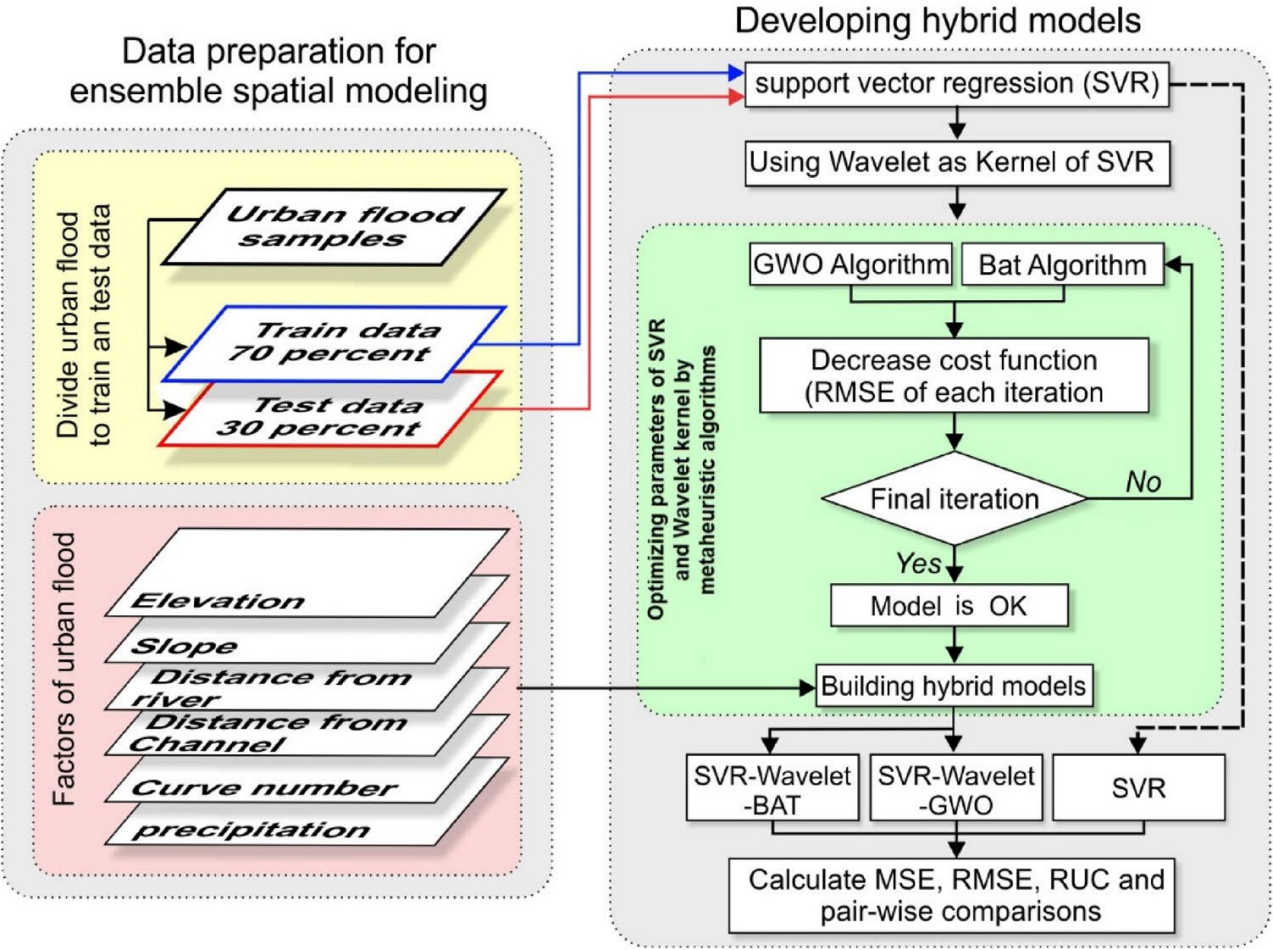
Flowchart of the methodology used in this study.

The methodological workflow developed in this study comprised the following steps (Fig. 10): preparation of dependent and independent variables, running the standalone SVR model, development of the two hybridized models (Wavelet-SVR-BAT and Wavelet-SVR-GWO), model validation, and comparison of model performance.

### Urban flood inventory

Historical flood inundation events in urban environments provide vital information for modeling. In this study, a total of 118 flood inundation locations were recorded by field investigations and available reports in the Amol Municipality (see Fig. 1). A ratio of 70:30 was used to randomly split the flood inventory dataset into two groups, for training (n = 83) and validation (n = 35) of the models.

### Factors influencing urban flood inundation

**Figure 11:**
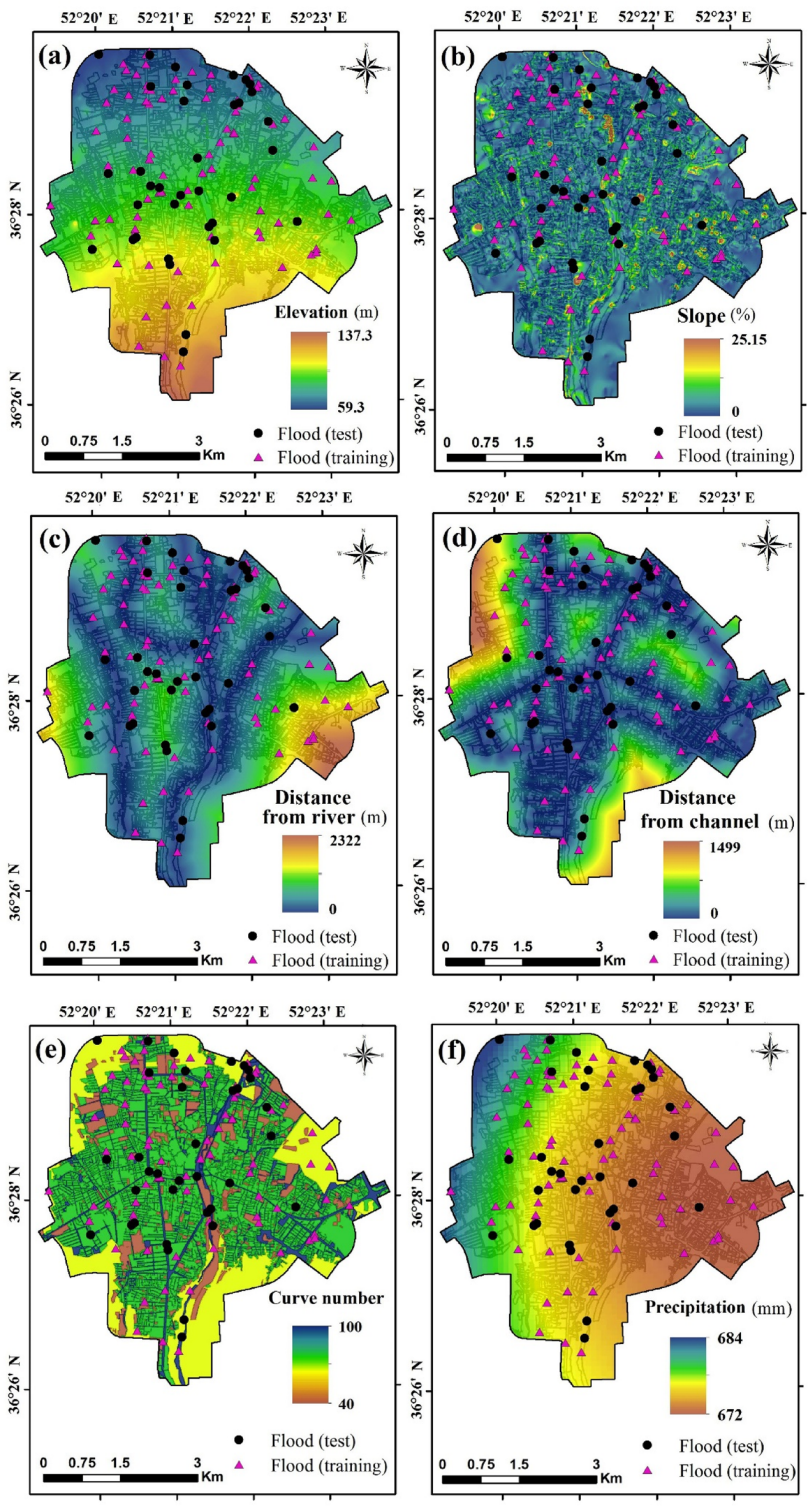
(**a**–**f**) Factors used to map flood conditioning in Amol city, and location of sites affected by previous floods and used for both training and validation of the models. These maps were generated using ArcGIS Desktop 10.7.1, https://desktop.arcgis.com/en/.

There are no universal guidelines for selecting factors that affect urban flood susceptibility, and previous researchers have considered different factors, depending on the model used and data availability. In this study, the selection of the flood conditioning factors was based on previous work by Darabi et al*.*^11^ and Falah et al.^23^ and data availability in the study region. The factors selected to map urban flood susceptibility in this study were elevation, slope percent, distance from river, distance from channel, curve number (CN), and precipitation (Fig. 11). River network was derived from an elevation layer map with 5-m spatial resolution provided by the Amol city authority. The elevation of Amol city ranges between 59 and 137 m (Fig. 11a). Slope as a topographical factor influences water flow characteristics such as velocity and discharge. It also impacts erosion and sedimentation processes. Lower slopes are generally seen in lowland areas of a watershed, which makes these susceptible to flooding^40^. Hence, slope could be an important factor in flood susceptibility investigations. Slope percent in Amol city varies from 0 to 25 (Fig. 11b). Rivers and channels are main paths of water flow in cities, where rivers are a natural feature, and channels are human-made. Major floods usually occur from rivers, but the inappropriate design of channels network can cause considerable flooding. Therefore, this study considered distance from rivers and channels and calculated these layers by Euclidean distance function. Distance from river in the Amol city ranges between 0 and 2,322 m (Fig. 11c), and distance from channel varies between 0 and 1,499 m (Fig. 11d). Curve number is an important variable that influences the permeability of soil or ground surface. This factor represents the impact of land use and soil permeability features. To create the CN layer, an ArcCN-runoff function was implemented. There is a wide range of CN in Amol city (40–100), which is high and shows a significant variation (Fig. 11e). Precipitation is the main source of water flow, especially for flooding. To take its impact into account, we acquired the precipitation map from the Mazandaran’s Forest, Range, and Watershed Management Organization. According to their report, 20 rain gauging stations were used for generating the precipitation map, based on the Kriging method due mainly to lower RMSE value compared with other interpolation methods such as inverse distance weighting (IDW). However, precipitation in the study area does not change dramatically, with a small spatial variation (672 to 684 mm year^−1^) in the study area (Fig. 11f). Further, the variance inflation factor (VIF) was calculated to investigate the multicollinearity between the factors and avoid bias in the results. A VIF value higher than 5 indicates a strong correlation between the factors and, accordingly, critical multicollinearity. Tolerance index, the reciprocal of VIF, was also used, with values lower than 0.2, indicating critical multicollinearity.

### Application of models

#### Support Vector Regression Algorithm

To solve regression issues and perform short-term forecasting, the support vector regression (SVR) algorithm, which is a supervised learning technique, can be applied^41^. The SVR algorithm, which is based on statistical learning theory, was first proposed by Vapnik^42^. This method can be employed to look for relationships between input and output data, based on structural risk minimization. In contrast, Neural Network Algorithms (NNA) and conventional statistical methods are based on empirical risk minimization. Thus the SVR method has superiority in reducing the generalization error as opposed to the learning error^43^. The purpose of SVR is to produce a function which explains the relationship between input and output data:

1 $$f(x) = w^{T} \psi (x) + b$$

where $x \in R^{n}$ denotes input data vector, $w \in R^{n}$ represents weight vector, $b \in R$ is a deviation, and $\psi (x)$ is a non-linear mapping function that transforms input data into high-dimensional feature space. Parameters $w$ and $b$ are based on the principle of structural risk minimization and are calculated as:

2 $$\begin{gathered} Minimize{:}\quad \left[ {\frac{1}{2}\left\| w \right\|^{2} + C\sum\limits_{i = 1}^{l} {\xi_{i} + \xi_{i}^{*} } } \right] \hfill \\ S.t.\left\{ \begin{gathered} y_{i} - w^{t} x_{i} - b \le \varepsilon + \xi_{i} \hfill \\ - y_{i} + w^{T} x_{i} + b \le \varepsilon + \xi_{i}^{*} \hfill \\ \xi_{i} ,\xi_{i}^{*} \ge 0 \hfill \\ \end{gathered} \right. \hfill \\ \end{gathered}$$

where $y_{i} \in R^{n}$ shows target data vector, $C \succ 0$ is a penalty factor determining the tradeoff between training error and model complexity, $\xi_{i} ,\xi_{i}^{*}$ represents slack variables which adjust the upper and lower constraints on the function $f(x)$, and $\varepsilon$ denotes the insensitive loss function, which represents the quality of approximation^43^. Equation 2 can be solved utilizing Lagrangian formulation and its final solution:

3 $$f(x) = \sum\limits_{i = 1}^{n} {(\alpha_{i} - \alpha_{i}^{*} )k(x} ,x_{i} ) + \, b$$

where $\alpha_{i} ,\alpha_{i}^{*}$ are Lagrangian multipliers and $k(x_{i} ,\;x_{j} ) = \langle \psi (x_{i} ),\;\psi (x_{j} )\rangle$ is the kernel function. Many different types of kernel function can be included (e.g., polynomial, Gaussian radial basis, exponential radial basis).

It should be noted that the SVR technique is highly dependent on model and forecast error to define its parameters, including penalty coefficient (*C*), permitted error range (ɛ), and kernel function parameters. For instance, the training error will be quite high if $C$ is at a very low level. On the contrary, if the penalty coefficient is very high, the learning accuracy will improve, but the general adoptions of forecasting models will be low in comparison with reality. When the number of supervised vectors is reduced on condition that $\varepsilon$ is high, the forecasting model is proportionately simple, but the accuracy will decline. On the other hand, if $\varepsilon$ is rather low, the complexity of the forecasting model increases, adoption decreases, and the accuracy of regression will be enhanced. To find the optimized value of these parameters, in this study, the Bat and Grey Wolf evolutionary optimization algorithms were applied^44^. A detailed description of the SVR algorithm can be found in Smola and Schölkopf^45^.

#### Translation invariant wavelet kernel

Kernel functions have been used in many different pattern analysis and ML techniques. These functions assist the SVR method in processing high-dimensional and infinite data, allowing linear separability by mapping input space data to higher dimensions. In addition, kernel functions keep the computational complexity of SVR reasonable in feature space^46^. Thus the number of support vectors and their weights, but also the type of kernel functions, are important and affect the results^47,48^. A wavelet function can be employed as the kernel function^49^. Generally, a wavelet function is defined as:

4 $$\psi_{a,b} (x) = \frac{1}{\sqrt a }\psi \left( {\frac{x - b}{a}} \right)\quad (a \succ 0,\;b \in R)$$

where $a$ and $b$ represent dilation (scaling) and translation (shift) parameters, respectively, $\psi (x)$ is the wavelet base or mother wavelet, and indexed families of wavelets are obtained by changing *a* and *b*^39,46^. If *a* and *b* are chosen on the basis of a power of 2 (*a* = 2^*j*^ and *b* = *k* × 2^*j*^), the discrete wavelet transform (DWT) is as follows^50^:

5 $$\psi_{j,k} (x) = \frac{1}{{2^{\frac{j}{2}} }}\psi \left( {\frac{x}{{2^{j} }} - k} \right)$$

where $k$ is a translation parameter and $j$ is an integer representing the resolution level, i.e., the dilation parameter. Based on the above, kernel function can be computed by inner products of wavelet function as:

6 $$k(X,X^{\prime}) = \langle \psi (X),\psi (X^{\prime})\rangle = \prod\limits_{i = 1}^{N} {\left( {\psi \left( {\frac{{x_{i} - b_{i} }}{a}} \right) \cdot \psi \left( {\frac{{x^{\prime}_{i} - b_{i}^{\prime } }}{a}} \right)} \right)} \quad a,\;b_{i} ,\;b_{i}^{\prime } ,\;x_{i} ,\;x^{\prime}_{i} \in R$$

where $X,X^{\prime} \in R^{N}$ are N-dimensional vectors and the translation-invariant wavelet kernel can be expressed as^51^:

7 $$k(X,X^{\prime}) = \prod\limits_{i = 1}^{N} {\psi \left( {\frac{{x_{i} - x^{\prime}_{i} }}{a}} \right)}$$

It should be pointed out that there are various wavelet functions, such as Haar, Splines, Daubechies, Marr, and Morlet. Gaussian function (Eq. 8) was used in this study due to its excellent reputation in terms of performance and possessing many useful features, as well as its strong learning capability^52–54^. Thus Eq. (7) was re-formulated as:

8 $$\psi_{Gaussian} (x) = \exp \left( {\frac{{ - x^{2} }}{2}} \right)$$

9 $$k(X,\;X^{\prime}) = \prod\limits_{i = 1}^{N} {\exp \left( { - \frac{{\left\| {x_{i} - x^{\prime}_{i} } \right\|}}{{2a^{2} }}^{2} } \right)}$$

Finally, the general form of the wavelet SVR (WSVR) regression function can be written as:

10 $$f(x) = \sum\limits_{i = 1}^{n} {(\alpha_{i} - \alpha_{i}^{*} )} \prod\limits_{j = 1}^{N} {\exp \left( {\frac{{ - \left\| {x_{j} - x^{\prime}_{j} } \right\|}}{{2a^{2} }}^{2} } \right)} + b$$

It should be noted that since WSVR has multidimensional analysis properties and high flexibility, and is a generalization of the SVR algorithm, it can be employed to solve non-linear problems and approximation and classification issues^46^. Studies have shown that the approximation effect of the wavelet kernel is far better than that of the Gaussian kernel^52^. Additionally, due to the redundancy and correlative features of the Gaussian kernel, the training speed of the wavelet kernel SVM is rather fast in comparison with that of the Gaussian kernel SVM^49^. More detailed explanations of wavelet SVR can be found in Zhang and Han^49^, and Su et al*.*^55^.

#### Bat Algorithm (BA)

In 2010, Xing-She Yang established the Bat metaheuristic algorithm, which derives from the echolocation characteristic of bats^35^. At night, bats can pinpoint their path and produce detailed images of their prey by comparing the emitting pulse with its echoes. In the Bat algorithm, the position of each bat indicates the potential solution and the quality of the solution is determined according to the best position of a bat to its prey. The approach was developed based on the following three rules^56^:

1. All bats apply echo sounds to recognize the distance and distinguish the dissimilarity between food/ prey and obstacles.
2. During the hunting period, bats fly unsystematically with certain velocity, at position $X_{i}$ with fixed frequency, $f_{\min }$, and varying wavelength, $\lambda$, and loudness, $A$. Bats can also automatically adjust wavelength and rate of pulse emission, $r \in [0,1]$, with regard to their distance to target.
3. Since loudness can vary in different directions, it can be assumed that the loudness changes from a maximum (positive) to a minimum constant value.

Like other metaheuristic algorithms, there is a well-suited tradeoff between the two main features, intensification (exploitation) and diversification (exploration), which guarantee that the Bat algorithm (BA) can assist in finding overall solutions^57^. According to the BA-SVR method, bats move within the parameter space and try to detect some optimized parameters. In other words, defining the optimal value for the SVR parameters is the main objective of implementing this approach^37^. For information, see Ali^58^, Sambariya et al*.*^59^, Yang^35^, and Yang and Gandomi^60^.

#### Grey wolf optimization (GWO) algorithm

The Grey Wolf Optimization (GWO) algorithm can be employed as another method to set optimum values of SVR parameters. The GWO algorithm was introduced by Mirjalili et al*.*^33^ as a new metaheuristic method. This technique is a bio-inspired global optimization algorithm and, like other metaheuristic methods, belongs to swarm intelligence approaches. The GWO algorithm is based on the leadership hierarchy and the social behavior of grey wolves at the time of hunting. Wolves are social animals that live in packs, and they have a hierarchy in their group. The leader of each pack, the alpha wolf ($\alpha$), dictates decisions about hunting, sleeping, and walking time, and all the other group members must follow its orders. However, despite the dominant manner of alpha wolves, democratic behavior can also be seen in the group. In terms of hierarchy, the other wolves fall into three levels, called beta ($\beta$), delta ($\delta$), and omega ($\omega$). The beta wolves at the second level assist the alpha in making decision. They are the best candidates for alpha replacement in due course. The delta wolves are in charge of defending the boundaries, warning the pack about any danger or threats to its territory, and ensuring the safety of the group. At the time of hunting or sourcing food, deltas help alpha and betas. The omega wolves have the lowest rank and rights in the group. They are not allowed to eat until all other wolves have finished eating, do not play any role in decision making, and in fact have a victim role^33^. However, in reality, theses wolves are a crucial part of the group, and their elimination can lead to fighting and serious social problems for the overall group^61^.

In addition to the social hierarchy of grey wolves, another remarkable characteristic is their group hunting, which can be summarized in three phases: (i) Tracking, chasing, and approaching prey, (ii) running after prey, surrounding, and harassing the prey up to the moment it stops running, and (iii) attacking and killing^62,63^.

In the GWO algorithm, mathematical modeling of wolves’ dominant social hierarchy behavior is performed by generating a random set of solutions, of which the solution with the best fit is considered the alpha ($\alpha$). The next best solutions are called beta ($\beta$) and delta ($\delta$), respectively, and remaining candidate solutions are called omega. The hunting process (optimization) is led by $\alpha$, $\beta$ , and $\delta$ wolves, and $\omega$ wolves have to comply with these three groups. The following four stages are performed: encircling, trapping and surrounding the prey, detecting prey location, attacking prey, and killing the prey (exploitation)^5^.

It is worth mentioning that GWO, as an optimization algorithm, has better search ability and higher accuracy than Genetic Algorithm (GA) and Particle Swarm Optimization (PSO)^33^. The GWO algorithm can be employed to find solutions to non-convex optimization problems. Its main advantages are its simplicity, ability to solve real-world optimization problems, and fewer control parameters^13^. Supplementary information can be found in Sulaiman et al*.*^64^, Niu et al*.*^65^, Jayakumar et al*.*^66^, Saxena et al*.*^34^, and Luo^51^.

### Accuracy assessment

In the present study, the prediction performance of the models was assessed by analyzing the agreement between observed data (flood inventory) and model results in terms of both presences (i.e., flooded locations) and absence (i.e., non-flooded locations)^11^. Three different evaluation approaches were used for assessing the accuracy of models in both the training (goodness-of-fit) and the validation (predictive performance) steps. These were cutoff-independent metrics (Sensitivity, Specificity, Accuracy, Matthews Correlation Coefficient, F-score, Misclassification Rate), cutoff-independent metrics (receiver operating characteristic (ROC) curve), and root mean square error (RMSE).

#### Cutoff-dependent metrics

All cutoff-dependent metrics were calculated based on a confusion matrix (also known as the contingency table). The components of the contingency table are true negative (TN), true positive (TP), false negative (FN), and false positive (FP) (Table 5), where FP and FN are the numbers of pixels erroneously classified (also known as error types I and II) and TN and TP are the numbers of pixels correctly classified. Note that a probability holdout value of 0.5 was chosen since the presence and absence locations were equally balanced, which is in accordance with Frattini et al*.*^67^ and Camilo et al*.*^68^.

**Table 5:** Contingency table used for evaluating models.

Observed	Predicted	
	Non-flooded (absence)	Flooded (presence)
Non-flooded (absence)	True negative (TN)	False positive (FP), Error type I
Flooded (presence)	False negative (FN), Error type II	True positive (TP)

##### True positive rate

True positive rate (TPR) (also termed sensitivity) is one of the most common evaluation metrics and can be calculated as:

11 $$TPR = \frac{TP}{{TP + FN}}$$

##### False positive rate

False positive rate (FPR) (also known as 1 − specificity) can be calculated as:

12 $$FPR = \frac{FP}{{FP + TN}}$$

However, it should be noted that TPR and FPR are insufficient performance metrics, because they ignore false positives (here the number of pixels erroneously identified as flooded) and false negatives (here the number of pixels erroneously identified as non-flooded). In fact, they are useful only when used together.

##### Accuracy

Accuracy (A, also known as efficiency) is another common metric for evaluating model accuracy. Accuracy determines the percentage of actual flooded points that are correctly classified by the model as:

13 $$A = \frac{TP + TN}{{TP + TN + FP + FN}}$$

##### Matthews correlation coefficient

The Matthews Correlation Coefficient (MCC) metric assesses the performance of models based on the correlation rate between observed and predicted data^69^. MCC ranges from − 1 to 1, where − 1 indicates considerable disagreement between observed and predicted data and 1 indicates perfect agreement. MCC is calculated as:

14 $$MCC = \frac{TP \times TN - FP \times FN}{{\left[ {\left( {TP + FP} \right) \times \left( {FN + TN} \right) \times \left( {FP + TN} \right) \times \left( {TP + FN} \right)} \right]^{{\left( {1/2} \right)}} }}$$

##### F-score

The F-score (also called the F1 score or F measure) is calculated as:

15 $$F\text{-}score = \frac{2TP}{{2TP + FP + FN}}$$

It can also be obtained based on the TPR and another evaluation metric, Positive Predictive Value (PPV), as:

16 $$F{ - }score = 2\frac{PPV \times TPR}{{PPV + TPR}}$$

where PPV is *TP/*(*TP* + *FP*).

##### Misclassification rate, MR

Misclassification rate considers both the false positive and false negative components and therefore reflects an overall error rate. MR can be computed as:

17 $$MR = \frac{FP + FN}{{FP + FN + TP + TN}}$$

#### Cutoff-independent metric

The area under the ROC curve (AUC) is the most important evaluation metric in natural hazard assessment^40^. The ROC curve simply plots the TPR (i.e., sensitivity) on the Y-axis against the FPR (i.e., 1 − specificity) on the X-axis. It is considered the real measure of model evaluation because it simultaneously includes all components of the confusion matrix and equitably estimates the overall quality of a model^67^. AUC is bounded by [0, 1]: the larger the AUC value, the better the performance of the model over the whole range of possible cutoffs. Based on the analytical expression for the ROC curve, denoted *f*, AUC can be calculated as:

18 $$AUC = \int_{0}^{1} {f\left( {{\text{FPR}}} \right) d {\text{FPR}} = 1 - \int_{0}^{1} {f^{ - 1} \left( {{\text{TPR}}} \right) d{\text{ TPR}}} }$$

#### Root mean square error

Root Mean Square Error (RMSE) is a frequently used measure of the agreement between observed and predicted values. In the present study, the RMSE was used to evaluate all models. It can be calculated as:

19 $$RMSE = \left[ {\frac{1}{N}\mathop \sum \limits_{i = 1}^{N} \left( {S_{i} - O_{i} } \right)} \right]^{1/2}$$

where *S*_*i*_ and *O*_*i*_ are observed and predicted values, respectively.

### Contribution of conditioning factors

The contribution of the flood conditioning factors (i.e., predictor variables) to the modeling process (relative importance) was investigated using a map-removal sensitivity analysis. The relative decrease (RD) in AUC values, which reflects the dependency of the model output on the conditioning factors, was calculated. RD can be calculated using the following equation:

20 $$RD = \frac{{AUC_{all} - AUC_{i} }}{{AUC_{all} }} \times 100$$

where AUC_all_ and AUC_i_ are the AUC values obtained from the flood susceptibility prediction using all conditioning factors and the prediction when the *i*th conditioning factor was excluded, respectively.
